# Association between SARS-CoV-2 levels in urban wastewater and reported COVID-19 cases in Changsha, Central China

**DOI:** 10.1186/s12879-025-11633-8

**Published:** 2025-10-09

**Authors:** Ling-Shuang Lv, Xiu-Ying Liu, Qian-Lai Sun, Heng Zhang, Jin-Fu Zhang, Zhi-Wen Yang, Ning-Yuan Guo, Xin Xia

**Affiliations:** 1 Hunan Provincial Center for Disease Control and Prevention, Hunan Academy of Preventive Medicine, Changsha, 410153 China; 2 Changsha Municipal Center for Disease Control and Prevention, Changsha, 410004 China

**Keywords:** Wastewater, SARS-CoV-2, COVID-19, Random forest model, Linear regression model

## Abstract

**Objective:**

To analyze the monitoring results of Severe Acute Respiratory Syndrome Coronavirus 2 (SARS-CoV-2) in urban wastewater, and explore the association between SARS-CoV-2 levels in urban wastewater and human Coronavirus disease 2019 (COVID-19) infection.

**Methods:**

The concentrations of SARS-CoV-2 RNA in urban wastewater and the number of reported COVID-19 cases were collected in Changsha, Central China, between March 22, 2023 and December 31, 2024. Correlation analysis was used to explore the correlation between the SARS-CoV-2 RNA levels in wastewater and the number of reported COVID-19 cases. Linear regression and random forest models were used to analyze the predictive function of SARS-CoV-2 in wastewater on human COVID-19 cases.

**Results:**

A total of 2,026 wastewater samples were collected. The positive rate of SARS-CoV-2 in wastewater was 82.0%. The positive rates of target Genes ORFlab and N were 71.7% and 81.4%, respectively. In the same period, 70,525 cases of COVID-19 were reported. The concentrations of target genes ORFlab and N in wastewater exhibited consistent trends (*r* = 0.95, 95% CI: 0.93–0.97) and were positively correlated with the number of reported COVID-19 cases (*r* = 0.79, 95% CI: 0.70–0.86; *r* = 0.77, 95% CI: 0.67–0.84). The linear regression model showed that the coefficients of determination between the number of reported COVID-19 cases and the levels of SARS-CoV-2 RNA (ORFlab, N) in wastewater were 0.63 and 0.59, respectively. In the test set, the random forest model indicated that the correlation coefficients between the number of reported COVID-19 cases and the predicted cases based on the concentration of target Gene ORFlab, target gene N and their combined concentration in wastewater were 0.89 (*R*^2^ = 0.77, *P* < 0.05), 0.88 (*R*^2^ = 0.75, *P* < 0.05) and 0.90 (*R*^2^ = 0.77, *P* < 0.05), respectively.

**Conclusions:**

Wastewater-based monitoring holds significant application value for trend prediction for COVID-19 cases.

**Supplementary Information:**

The online version contains supplementary material available at 10.1186/s12879-025-11633-8.

## Background

The emergence of COVID-19, caused by SARS-CoV-2, has posed an unprecedented threat to global public Health since 2019 [1]. As of February 2025, the WHO reported 780 million confirmed infections and 7.088 million deaths worldwide [2, 3]. Over the three-year pandemic period, the global response to the pandemic has evolved significantly, with advancements in vaccines, treatments, and public health measures [4]. Notably, the disease trajectory has shifted from acute pandemic to endemic equilibrium [5]. In response to this epidemiological shift, on January 8, 2023, China implemented a significant policy adjustment. It began to manage the relevant situation as a Class B infectious disease instead of a Class A infectious disease [6].

At present, the management of COVID-19 has transitioned to a normalized stage. Given the cessation of comprehensive large-scale nucleic acid-testing, public health systems are adapting their approaches to monitoring SARS-CoV-2 transmission patterns [4]. Current surveillance, while primarily based on clinical testing that may not capture all cases, is complemented by community-level indicators such as laboratory test positivity rates and wastewater surveillance, as well as severe disease metrics including hospitalization and mortality rates [7–9]. Although respiratory transmission via aerosol and droplet inhalation, remains the primary route of SARS-CoV-2 transmission [10, 11]. The increasing evidence highlights wastewater-based epidemiology as a critical complementary approach for pandemic surveillance [12, 13]. This approach leverages wastewater as a naturally pooled community sample, providing population-level health data that enables viral strain characterization with minimal sampling requirements [14]. Crucially, wastewater captures the complete spectrum of infections, including both symptomatic and asymptomatic cases, regardless of clinical testing status. Detecting SARS-CoV-2 in untreated wastewater can be a useful tool to identify early outbreak, transmission trend, and community prevalence [15]. Compared to population-wide nucleic acid testing, wastewater surveillance offers substantial advantages in cost-efficiency and resource utilization. The method's non-invasive nature eliminates individual testing requirements while generating aggregated data that preserves personal privacy, making it particularly valuable for ongoing public health monitoring.

The first study of SARS-CoV-2 wastewater surveillance was conducted in the Netherlands, where viral RNA was detected in wastewater treatment influent samples in seven major cities and the international airport [16]. Subsequent studies in the United States [17] and Canada [18] further validated the utility of wastewater-based epidemiology in COVID-19 surveillance. Up to now, 72 countries and regions had established National Wastewater Surveillance Systems to help public health officials better assess COVID-19 prevalence in communities [19]. Previous study demonstrated a correlation between SARS-CoV-2 RNA concentrations in municipal wastewater and COVID-19 cases (median *r* = 0.66) [20]. A systematic review and meta-analysis investigating the association between SARS-CoV-2 RNA concentration in wastewater and community COVID-19 cases identified 133 correlation coefficients (range: −0.38 to 0.99), with substantial variability across studies [21]. This variability may be attributed to differences in environmental conditions, epidemiological contexts, and sampling methodologies across the included studies.

Wastewater surveillance has become a critical component of the infectious disease surveillance and early warning system in China [22]. Nevertheless, significant inconsistencies persist in the correlation between wastewater SARS-CoV-2 RNA concentrations and clinically reported COVID-19 cases across different areas [21, 23]. To evaluate the effectiveness of urban wastewater surveillance in predicting COVID-19 prevalence, we collected and analyzed the surveillance data of SARS-CoV-2 RNA in wastewater and COVID-19 cases from Changsha, Central China, during March 22, 2023 to December 31, 2024.

## Methods

### Study location

This study was conducted in Changsha, the capital of Hunan Province in Central China. Changsha is located between 27°51′−28°40′N and 111°53′−114°15′E, covers an area of 11,819 km^2^, and has a population of more than 10 million. Changsha is located in the lower reaches of the Xiangjiang River, which runs through the urban area. The surveillance period spanned from March 22, 2023 (Week 12) to December 31, 2024 (Week 52).

### Wastewater samples collection, preservation and transportation

The wastewater surveillance data was sourced from the Chinese Urban Wastewater Surveillance System (CWSS). During the study period, a total of 2,026 wastewater samples were collected. Wastewater samples were collected using refrigerated (4 °C) automatic samplers equipped with self-cleaning functions at the influent channels of 12 municipal wastewater treatment plants. Twenty-four-hour composite samples were Generated through hourly sampling intervals, with 500 mL aliquots collected from mixed samples. All samples were immediately placed in containers maintained at 0–4 °C and transported to the laboratory within 2 h. The sampling frequency was set as biweekly from Week 20, 2023, to Week 44, 2024, and weekly during the remaining weeks. The data of daily wastewater treatment volume (WTV), inlet water temperature, suspended solids, chemical oxygen demand (CODCr), pH, and ammonia nitrogen were collected. All of the methods refer to the Technical Manual of Pathogen Monitoring in Municipal Sewage (2024 edition).

### The test method of wastewater samples

The sealed sample bottles were fully submerged in a 60 °C water bath and incubated for 30 min to inactivate SARS-CoV-2. Viral enrichment, concentration, and nucleic acid detection were performed following the National Health Commission of China Standard WS/T 799–2022: Method for enrichment and nucleic acid detection of SARS-CoV-2 in wastewater [24]. The aluminum salt coagulation precipitation method was used for wastewater enrichment. Total nucleic acid was extracted from enriched wastewater samples, and subsequently subjected to fluorescent quantitative reverse transcription polymerase chain reaction (RT-qPCR). Amplification was performed on a real-time PCR system, with results analyzed based on amplification curves and kit-specific interpretation criteria. Standard curves were made for RT-qPCR of each batch of samples. The standard curve was plotted with the average Ct value of the standard substance at the same concentration as the vertical coordinate and the logarithm of the concentration as the horizontal coordinate. The amplification efficiency and coefficient of determination were obtained. When the amplification efficiency is within the range of 90% to 110% and coefficient of determination ≥ 0.99, the standard curve is valid. The average Ct value of target genes ORF1ab and N was substituted into the standard curve to calculate the concentration of SARS-CoV-2 in the sample, with the unit of copies/mL.

### The data of COVID-19 cases

The Daily data of COVID-19 cases in Changsha in same period were obtained from the China Disease Prevention and Control Information System. The weekly COVID-19 cases were calculated based on daily COVID-19 report data.

### Statistical analysis

Previous studies used a 5-day moving average of reported cases, as SARS-CoV-2 can persist in the secretions of infected individuals for more than two weeks [25]. In our study, as the wastewater is monitored weekly, we calculated the 7-days average of reported cases. The positive rate of SARS-CoV-2 in wastewater is the proportion of positive results in the SARS-CoV-2 RNA detection at all monitoring points during each week. The weekly weighted average concentrations of SARS-CoV-2 RNA (target genes ORF1ab and N) in wastewater were calculated. The weighted average concentration refers to the sum of SARS-CoV-2 RNA concentration and wastewater flow of each monitoring point in a given week divided by the sum of the daily wastewater flow of each monitoring point. The SARS-CoV-2 surveillance data in wastewater and the reported number of COVID-19 cases were matched according to the data of year and week.

Data collation and analysis were carried out using R software (version 4.3.3). Pearson's correlation analysis was performed to explore the correlation between SARS-CoV-2 RNA concentrations in wastewater and the number of reported COVID-19 cases. Linear regression model was used to analyze the fitting effect. In linear regression model, *x* represents target gene ORF1ab or N concentration in wasterwater (copies/mL), and *y* represents reported COVID-19 cases (cases/week). According to the linear fitting formula, we used the average concentration of SARS-CoV-2 RNA in wastewater per week to calculate the predicted value of the weekly average COVID-19 cases. Given that the target gene ORF1ab and N concentration in wasterwater exhibit collinearity, we further supplemented our analysis with a random forest model. According to the random forest model, we first adopted a random partitioning strategy: 70% of the data was randomly selected as the training set and the remaining 30% as the test set [26, 27]. A fixed random seed was set to ensure reproducibility of this random split. In addition, we employed a time-series-based partitioning method to further validate the model: the first 70% of the data (by chronological order) was designated as the training set, with the subsequent 30% used as the test set. In the random forest model, the reported number of COVID-19 cases was set as the dependent variable, while the concentration of SARS-CoV-2 RNA in wastewater and the physicochemical detection indicators of wastewater were the independent variables. Subsequently, we compared the number of COVID-19 cases predicted by the random forest model with the actually reported values. R^2^ was used to represent the fitting effect between the predicted value and the actual value [28]. If R^2^ ≥ 0.9, it indicates an extremely strong fit. If 0.7 ≤ R^2^ < 0.9, it indicates a strong fit. If 0.5 ≤ R^2^ < 0.7, it indicates a moderate fit. If R^2^ < 0.5, it indicates a weak fit or no fit. *P* ≤ 0.05 is considered statistically significant.

## Results

### Basic information of wastewater

The daily average value of WTV during the sampling period was 177,000 tons/day, ranging from 23,500 tons/day to 705,700 tons/day. The daily average values of inlet water temperature, total suspended solids, CODCr, pH and ammonia nitrogen were 21.8 °C, 197.2 mg/L, 192.5 mg/L, 7.3 and 16.7 mg/L respectively (Table 1).

**Table 1:** Basic physicochemical information of wastewater monitoring

Variables	Mean	SD	P_5_	P_25_	P_50_	P_75_	P_95_
WTV (10,000 tons/day)	17.7	13.7	3.7	7.8	13.4	24.0	46.3
Inlet water temperature (°C)	21.8	5.6	12.0	180	22.5	26.2	29.3
Total suspended solids (mg/L)	197.2	155.5	69.0	108.0	156.0	236.0	420.8
CODCr (mg/L)	192.5	96.7	82.0	127.0	171.0	237.8	362.8
pH	7.3	0.2	6.9	7.2	7.34	7.5	7.7
Ammonia nitrogen (mg/L)	16.7	6.0	6.6	12.6	17	20.8	26.4

### The positive rate of SARS-CoV-2 RNA in wastewater

**Fig. 1:**
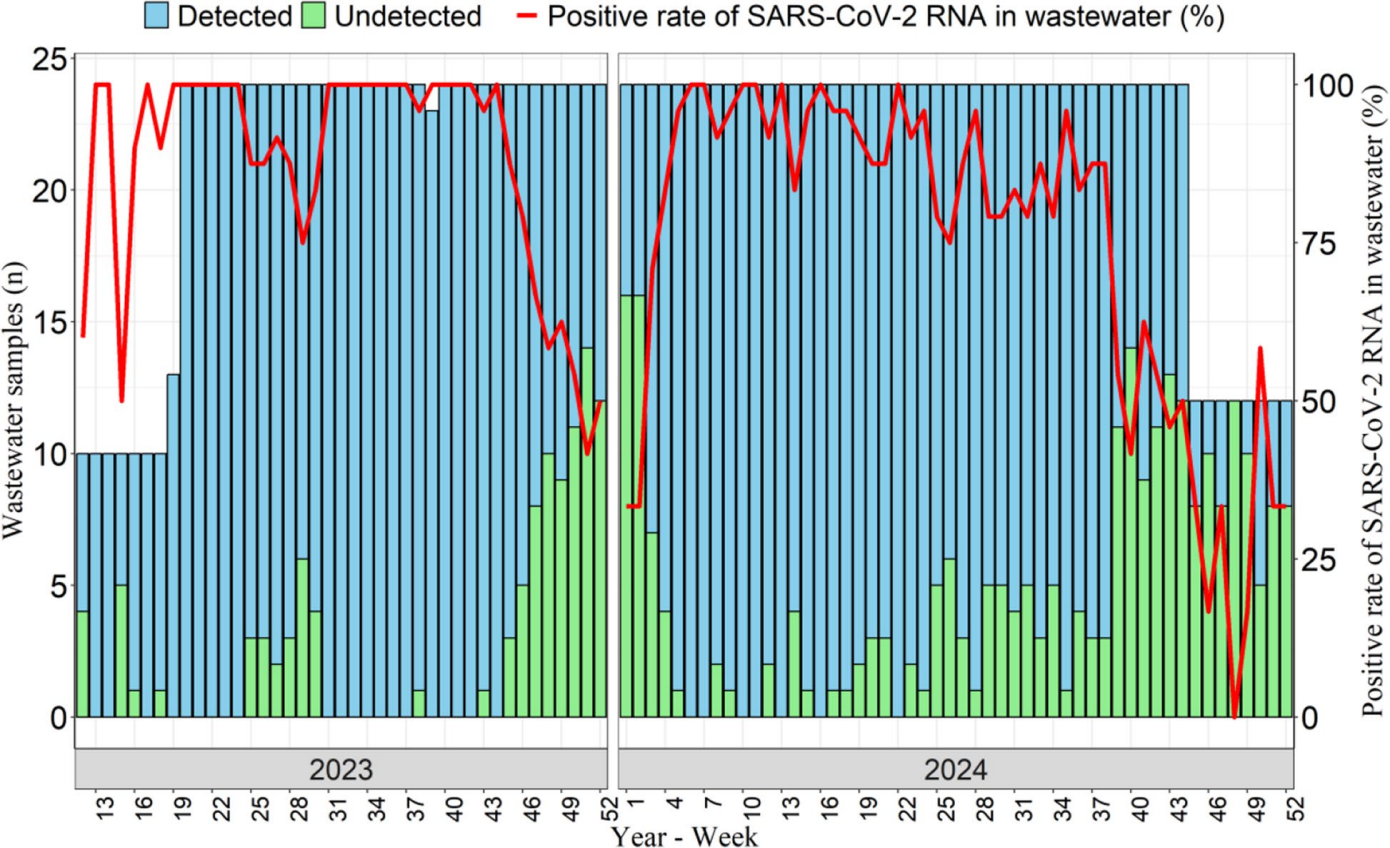
Dynamics of positive rate of SARS-CoV-2 RNA in wastewater in Changsha during 2023–2024. The blue and green vertical bars denote the number of detected or undetected SARS-CoV-2 RNA in wastewater in a given week. The red line denotes the positive rate of SARS-CoV-2 RNA in wastewater (%)

A total of 2,026 samples were collected, with 874 samples in 2023 and 1,152 samples in 2024, respectively. The positive rate of SARS-CoV-2 RNA in wastewater was 82.0%. The positive rates in 2023 and 2024 were 87.9% and 77.5%, respectively. In terms of the temporal distribution, from Week 16 to Week 45 in 2023 and from Week 5 to Week 38 in 2024, the positive rates of SARS-CoV-2 RNA in wastewater remained at a relatively high level (Fig. 1).

### The association between concentration of SARS-CoV-2 RNA in wastewater and the reported number of COVID-19 cases

**Fig. 2:**
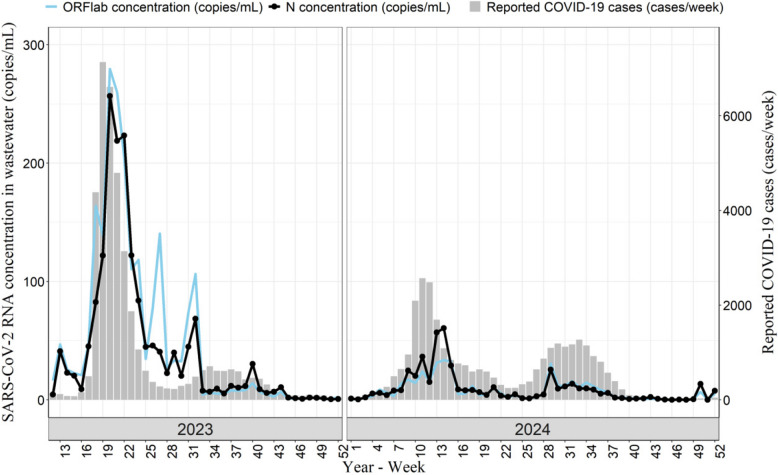
Dynamics of SARS-CoV-2 RNA concentrations in wastewater and the reported COVID-19 cases in Changsha during 2023–2024. The blue line denotes the mean concentration of target gene ORFlab in wastewater per week. The black plot denotes the mean concentration of target gene N in wastewater per week. The gray vertical bars denote the reported COVID-19 cases in a given week

The detection rates of the target genes ORFlab and N were 71.7% and 81.4%, respectively. The average concentration of the target Gene ORFlab was 23.72 copies/mL. Specifically, it was 41.98 copies/mL in 2023 and 9.89 copies/mL in 2024. The average concentration of the target Gene N was 26.72 copies/mL. Specifically, it was 50.59 copies/mL in 2023 and 8.61 copies/mL in 2024. The reported number of COVID-19 cases was 70,525, among which 39,467 cases were reported in 2023 and 31,058 cases were reported in 2024. The variation trends of the concentration of the target genes ORFlab and N in wastewater were relatively consistent with those of the reported number of COVID-19 cases (Fig. 2). The correlation analysis showed that there was a positive correlation between the concentration of target genes ORFlab and N (*r* = 0.95, 95% CI: 0.93–0.97, *P* < 0.05). Positive correlations were observed between the concentrations of target genes ORFlab and N and the reported number of COVID-19 cases. For the correlation with target Gene ORFlab, the correlation coefficient was 0.79 (*P* < 0.05, 95% CI: 0.70–0.86), while for target Gene N, the correlation coefficient was 0.77 (*P* < 0.05, 95% CI: 0.67–0.84).

### Prediction by the linear regression model

**Fig. 3:**
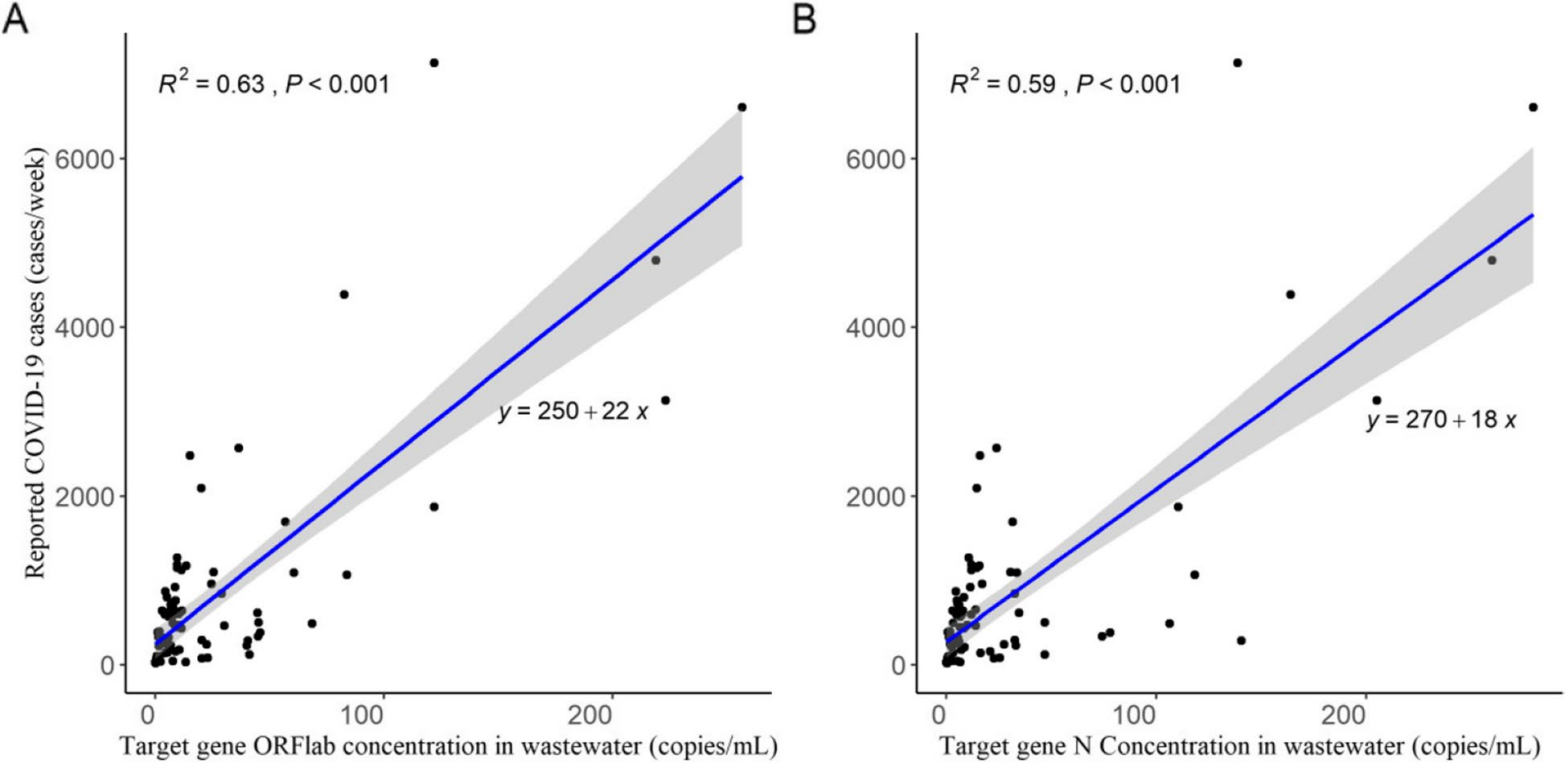
Linear fitting between SARS-CoV-2 RNA in wastewater and and the reported weekly COVID-19 cases in Changsha during periods 2023–2024. **A** The association between concentration of target gene ORFlab in wastewater and the reported weekly COVID-19 cases. **B** The association between concentration of target gene N in wastewater and the reported weekly COVID-19 cases

**Fig. 4:**
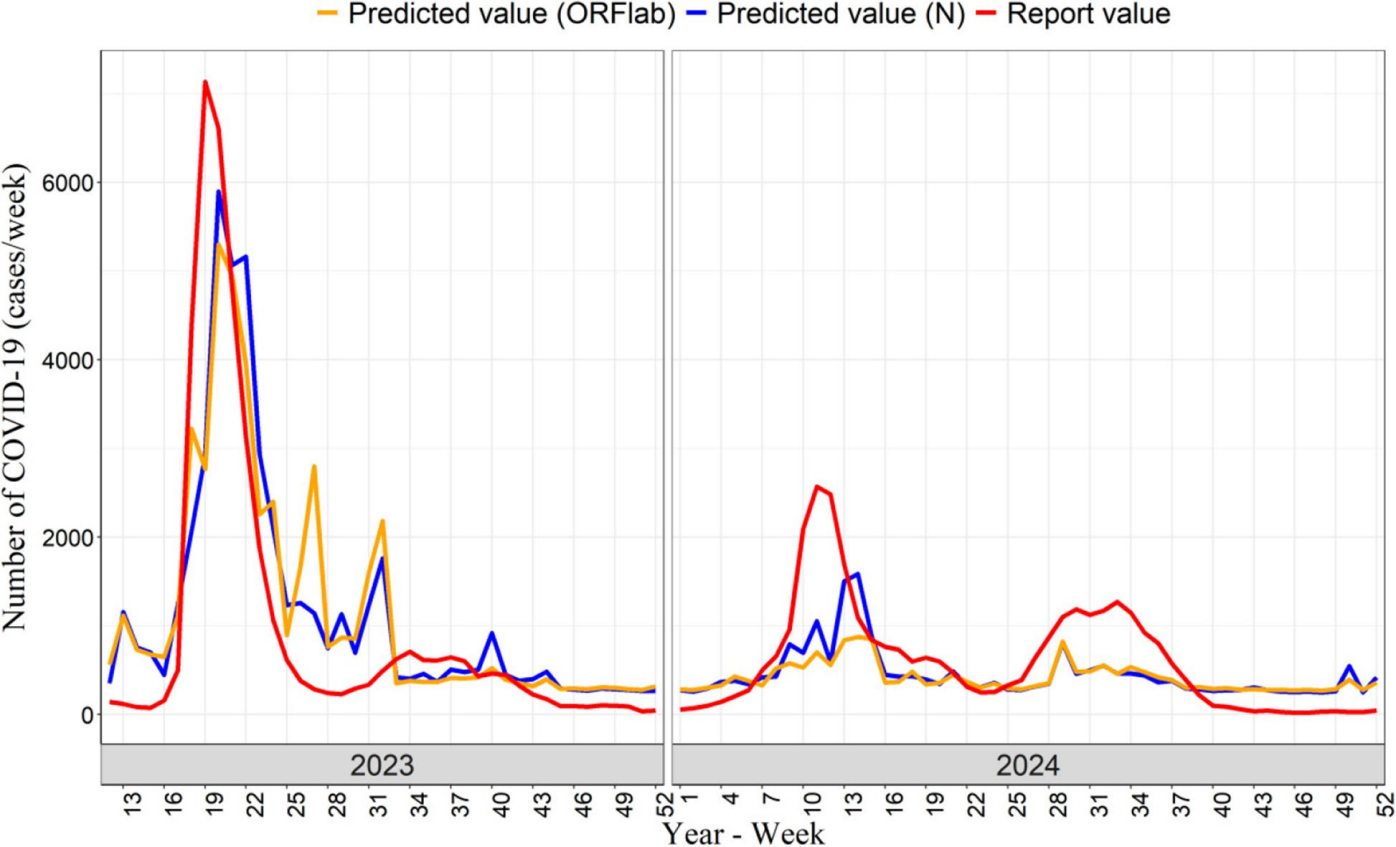
Variation trends of the weekly average reported values and predicted values of the number of COVID-19 cases. The red line represents the reported values of COVID-19 cases. The blue line denotes the predicted values of COVID-19 cases based on target gene ORFlab. The orange line indicates the predicted values of COVID-19 cases obtained from target gene N

The linear regression model was used to analyze the relationship between the concentration of SARS-CoV-2 RNA in wastewater and the reported number of COVID-19 cases (Fig. 3A, B). For target gene ORFlab, the linear fitting formula was *y* = 250 + 22*x*, with the R^2^ values of 0.63 (*P* < 0.001). For target gene N, the linear fitting formula was *y* = 270 + 18*x*, with the R^2^ values of 0.59 (*P* < 0.001). Comprehensive regression diagnostics are presented in Supplementary Fig. 1. According to the linear fitting equation combined with the concentration of SARS-CoV-2 RNA in wastewater, the predicted values of COVID-19 cases were calculated. The variation trends of the predicted values and the reported values of the weekly average number of COVID-19 cases were shown in Fig. 4. In terms of the prediction trend, the predicted values and the reported values of COVID-19 cases exhibited the same increasing and decreasing trends. Regarding the prediction of the epidemic peaks, both the reported values and the predicted values showed three peaks. When it comes to predicting the number of COVID-19 cases, the predicted values were relatively close to the reported values. However, the peak value of the predicted values are lower than that of the reported values.

### Prediction by the random forest model

**Fig. 5:**
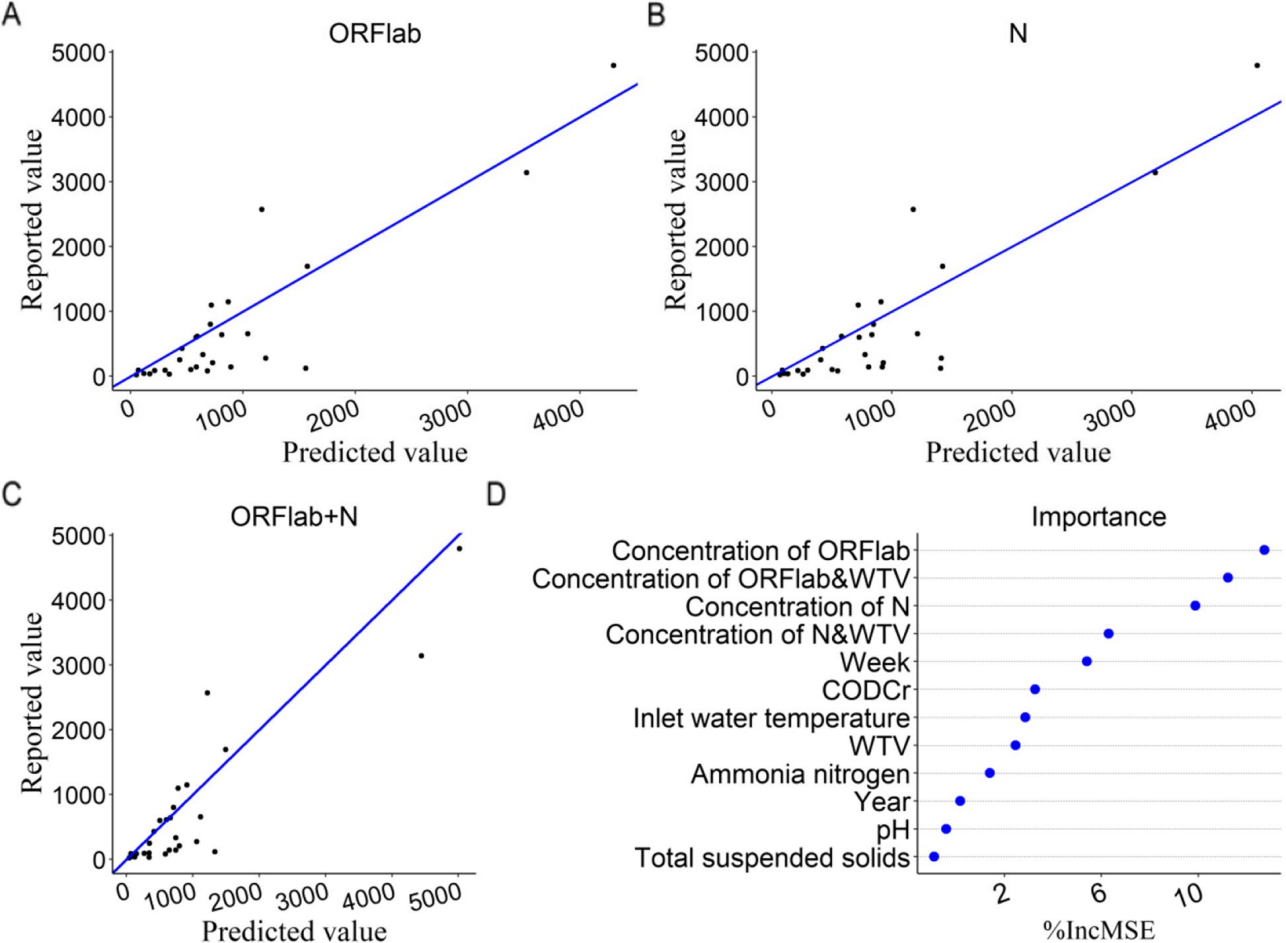
Correlation between the weekly reported values and predicted values of COVID-19 cases in the test set. **A** The relationship between the reported values and predicted values of COVID-19 cases based on the concentration of target gene ORFlab in wastewater. **B** The association between the reported values and predicted values of COVID-19 cases obtained from the concentration of target gene N in wastewater. **C** The relationship between the reported values and predicted values of COVID-19 cases derived from the combined concentrations of target genes ORFlab and N in wastewater. **D** Feature importance analysis of random forest model

Furthermore, the random forest model was used to analyze the relationship between SARS-CoV-2 RNA in wastewater and the reported number of COVID-19 cases. For the model parameters, the number of trees was set to 800, and the number of features randomly selected at each node was set to 4. The model performance metrics of the random forest are presented in Supplementary Table 1. The correlation coefficients between the reported number of COVID-19 cases and the predicted cases based on the concentration of the target Genes ORFlab and N, and the combined concentration of the two, were 0.89 (*R*^2^ = 0.77), 0.88 (*R*^2^ = 0.75) and 0.90 (*R*^2^ = 0.77), respectively (Fig. 5A, B, C). The feature importance analysis showed that the main variables affecting the prediction effect of the model were the concentration of the target genes ORFlab and N. Among the physicochemical indexes of wastewater, CODCr, inlet water temperature and WTV had a certain influence on the prediction results of the model (Fig. 5D). Correlation between the weekly reported values and predicted values of COVID-19 cases in the test set based on time series are presented in Supplementary Fig. 2.

## Discussions

Wastewater monitoring is an emerging topic in public health. As an "underground scout" for health risks, it offers a unique perspective on population health surveillance. This study monitored the SARS-CoV-2 in urban wastewater and COVID-19 cases in the population of Changsha, aiming to evaluate the application value of wastewater monitoring in epidemic prevention and control. The results showed that the positive rate of SARS-CoV-2 RNA in wastewater was relatively high, reaching 82.0%. Specifically, the rates were 87.8% and 77.5% in 2023 and 2024, respectively. The positive rate was higher than some other studies [29, 30]. A meta-analysis conducted in 2024 reported that the overall positive rate of SARS-CoV-2 RNA in municipal wastewater samples was 53.7%, with significant regional variations [29]. Another meta-analysis conducted in 2021 reported that, across 26 countries worldwide, the proportion of positive samples in wastewater ranged from 11.6% to 100% [15]. This discrepancy might be attributable to factors such as the population infection rate, the enrichment efficiency of SARS-CoV-2 RNA in wastewater, and the sensitivity of the detection method.

Previous studies have mostly focused on developed regions with mature centralized sewerage infrastructure, such as the Netherlands [31], USA [32]. In China, Chinese Urban Wastewater Surveillance System (CWSS) represents a brand-new project, which was officially initiated in December 2022. This study has obtained the first batch of data resources in this field, and the data exhibit remarkable timeliness and cutting-edge characteristics. Changsha, as a representative of medium-developed "new first-tier cities", features more universal urban scale, population structure and pipeline characteristics. It can provide more referential localized data for wastewater monitoring in similar cities. The study period was from 2023 to 2024, falling into the phase of normalized COVID-19 prevention and control. Wastewater monitoring conducted during this period enables more objective reflection of occult SARS-CoV-2 transmission dynamics in post-pandemic communities. Concurrently, it clarifies the correlation between SARS-CoV-2 viral loads in wastewater and human COVID-19 infections, while evaluating the utility of normalized monitoring systems in assessing epidemic fluctuation risks.

The correlation analysis showed that there were positive correlations between the concentration of the target genes ORFlab and N and the reported number of COVID-19 cases. The increasing and decreasing trends of the concentration of SARS-CoV-2 RNA in wastewater and the number of COVID-19 cases were relatively consistent, which were similar to previous results [33–35]. In the linear regression model, the R^2^ values were 0.63 and 0.59 for target genes ORFlab and N, respectively. Based on the linear regression model, the trends of variation in the predicted values for the weekly COVID-19 cases were similar to those of the reported values. However, the predicted values were generally lower than the reported values. Wastewater monitoring can largely reflect ‘true’ infection rate, including both tested and untested patients [36]. Nevertheless, there is a certain degradation of SARS-CoV-2 RNA in wastewater [37]. Thus, our results align with the European Commission’s recommendation, which emphasizes that wastewater surveillance serves as a tool for observing trends rather than an absolute means of determining the prevalence of COVID-19 in the population [38]. Given that the linear regression relies on only a single independent variable, its predictive ability is restricted. Consequently, more variables may be included to optimize the model fitting effect.

Previous studies have shown that there is a correlation between the concentration of SARS-CoV-2 RNA and the relevant physicochemical indexes of wastewater (pH, electrical conductivity, five-day biochemical oxygen demand, chemical oxygen demand, total nitrogen, total phosphorus, total suspended solids) [39], which provides a potential direction for further improving the model. As one of the most common integrated machine learning methods, the random forest model improves the prediction performance by constructing multiple decision trees, especially suitable for dealing with smaller data sets [26, 40]. In this study, SARS-CoV-2 RNA in wastewater and relevant physicochemical indexes were included in the random forest model. The results showed that the R^2^ value of the model was between 0.75 and 0.77, indicating that SARS-CoV-2 RNA in wastewater had a strong explanatory ability for COVID-19 cases. This result suggested that wastewater monitoring can effectively estimate the number and change trend of population infections. The feature importance analysis showed that the concentration of the target gene ORFlab played a more prominent role in the model. However, in actual monitoring, the positive rate of target gene ORFlab was lower than that of target gene N. Previous study evaluated three different gene targets of SARS-CoV-2, and highlights the importance of using multiple viral targets to ensure robust and reliable detection and quantification of SARS-CoV-2 in wastewater surveillance [41]. Moreover, the use of multiple viral gene targets has been shown to improve the reliability of SARS-CoV-2 surveillance in wastewater over time [37, 42]. Therefore, to improve the accuracy and reliability of the prediction, this study recommends comprehensively predicting the number of COVID-19 cases by integrating the concentration target genes ORFlab and N of SARS-CoV-2 in wastewater.

This study has certain limitations. First, due to the complexity of wastewater, the concentration of SARS-CoV-2 RNA were subject to certain uncertainties. This study did not account for the virus survival, persistence and degradation in the wastewater. Second, the pre-, during and post-symptomatic stages of COVID-19 cases were not considered. Moreover, the case number of COVID-19 were counted based on residential address, without considering the impact of commuting. Third, the data mainly came from the monitoring under the background of normalized epidemic prevention and control, and lacked the data during the most severe period of the COVID-19 epidemic from 2020 to 2022, which may affect the predictive ability of the model under extreme epidemic conditions. Forth, since the monitoring frequency was 1 to 2 times a week, only the weekly average value of the number of COVID-19 infections could be predicted based on the weekly average value of the concentration of SARS-CoV-2 RNA in wastewater. This low-frequency monitoring limited our in-depth discussion of the dynamic association between SARS-CoV-2 in wastewater and population infections, and also had a certain limitation on the practical application of the model fitting effect and prediction results.

## Conclusions

This study indicated that wastewater monitoring has important application value in the prevention and control of COVID-19 infection epidemics. By rationally utilizing the results of wastewater monitoring, it can provide a scientific basis for the early warning of epidemic rebounds and the formulation of prevention and control strategies. This approach will enable the full realization of its potential in epidemic prevention and control, thereby offering more comprehensive and accurate support for public health decision-making. Therefore, the widespread application of wastewater monitoring should be further optimized and strengthened, especially the verification and optimization in different regions and different epidemic backgrounds.

## Supplementary Information

Supplementary Figure 1. Comprehensive regression diagnostics. Supplementary Figure 2. Correlation between the weekly reported values and predicted values of COVID-19 cases in the test set based on time series. Supplementary Table 1. The model performance metrics of the random forest.

## Data Availability

All data can be obtained from the corresponding author upon request.

## Abbreviations

SARS-CoV-2: Severe Acute Respiratory Syndrome Coronavirus 2
COVID-19: Coronavirus disease 2019
WTV: Wastewater treatment volume
CODCr: Chemical oxygen demand
RT-qPCR: Reverse transcription polymerase chain reaction
R^2^: Coefficient of determination
