# Supplementary material for: Association between SARS-CoV-2 levels in urban wastewater and reported COVID-19 cases in Changsha, Central China
Source: BMC Infect Dis. 2025 Oct 9;25:1256. doi: 10.1186/s12879-025-11633-8 (PMC12512251; doi:10.1186/s12879-025-11633-8)
Supplement: Supplementary file 1 — Supplementary Figure 1. Comprehensive regression diagnostics. Supplementary Figure 2. Correlation between the weekly reported values and predicted values of COVID-19 cases in the test set based on time series. Supplementary Table 1. The model performance metrics of the random forest. [file 12879_2025_11633_MOESM1_ESM.docx]

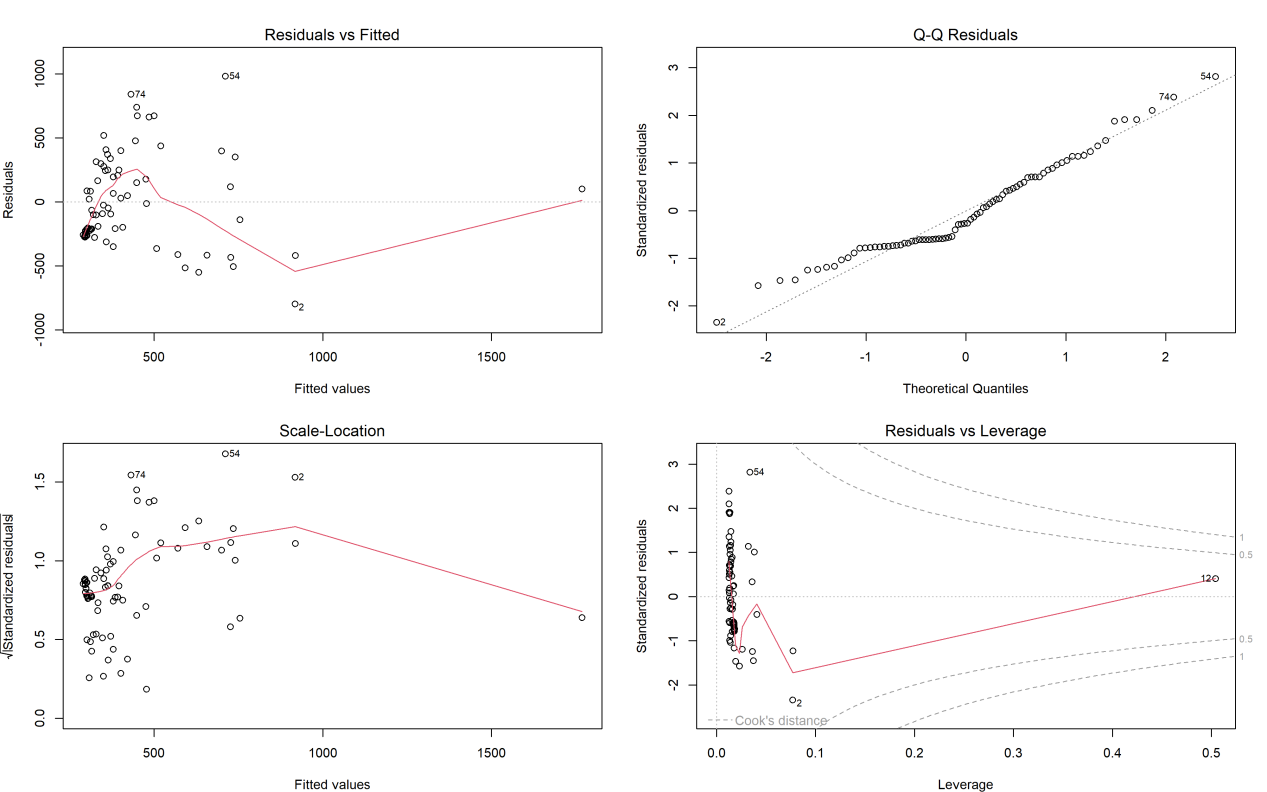


Supplementary Figure 1. Comprehensive regression diagnostics.


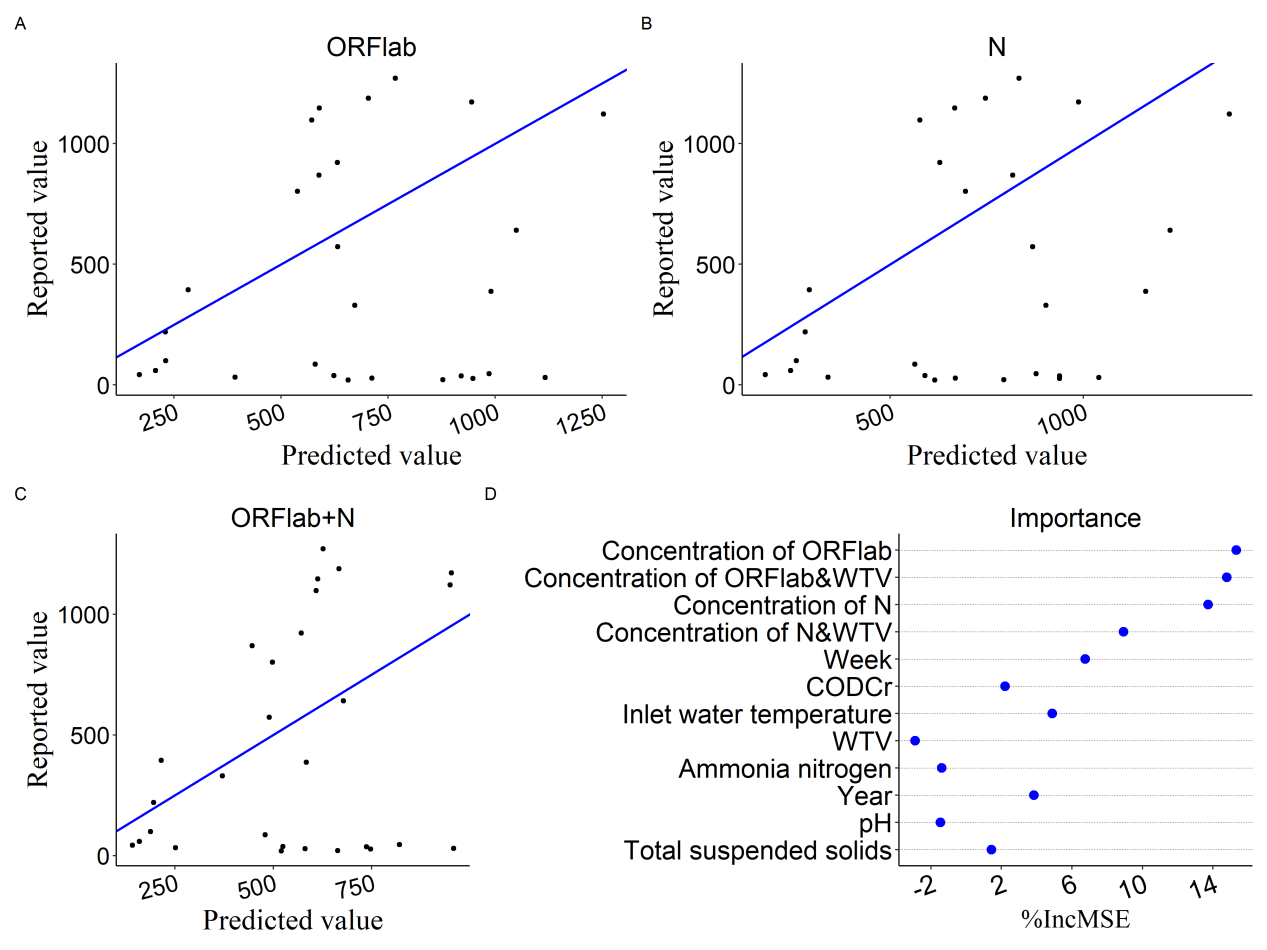


Supplementary Figure 2. Correlation between the weekly reported values and predicted values of COVID-19 cases in the test set based on time series.

Supplementary Table 1. The model performance metrics of the random forest.

| Evaluation indicators | Test dataset | Train dataset |
| --- | --- | --- |
| Model 1 (Target ORFlab) |  |  |
| RMSE | 504 | 512 |
| R^2^ | 0.68 | 0.85 |
| Model 2 (Target N) |  |  |
| RMSE | 434 | 516 |
| R^2^ | 0.76 | 0.85 |
| Model 3 (Target ORFlab + N) |  |  |
| RMSE | 371 | 492 |
| R^2^ | 0.83 | 0.87 |
| Notes: RMSE is Root Mean Square Error. R^2^ is the coefficient of determination | | |
